# Achieving accurate estimates of fetal gestational age and personalised predictions of fetal growth based on data from an international prospective cohort study: a population-based machine learning study

**DOI:** 10.1016/S2589-7500(20)30131-X

**Published:** 2020-06-23

**Authors:** Russell Fung, Jose Villar, Ali Dashti, Leila Cheikh Ismail, Eleonora Staines-Urias, Eric O Ohuma, Laurent J Salomon, Cesar G Victora, Fernando C Barros, Ann Lambert, Maria Carvalho, Yasmin A Jaffer, J Alison Noble, Michael G Gravett, Manorama Purwar, Ruyan Pang, Enrico Bertino, Shama Munim, Aung Myat Min, Rose McGready, Shane A Norris, Zulfiqar A Bhutta, Stephen H Kennedy, Aris T Papageorghiou, Abbas Ourmazd, International Fetal and Newborn Growth Consortium for the 21st Century (INTERGROWTH-21st), S Norris, SE Abbott, A Abubakar, J Acedo, I Ahmed, F Al-Aamri, J Al-Abduwani, J Al-Abri, D Alam, E Albernaz, H Algren, F Al-Habsi, M Alija, H Al-Jabri, H Al-Lawatiya, B Al-Rashidiya, DG Altman, WK Al-Zadjali, HF Andersen, L Aranzeta, S Ash, M Baricco, FC Barros, H Barsosio, C Batiuk, M Batra, J Berkley, E Bertino, MK Bhan, BA Bhat, ZA Bhutta, I Blakey, S Bornemeier, A Bradman, M Buckle, O Burnham, F Burton, A Capp, VI Cararra, R Carew, VI Carrara, AA Carter, M Carvalho, P Chamberlain, Ismail L Cheikh, L Cheikh Ismail, A Choudhary, S Choudhary, WC Chumlea, C Condon, LA Corra, C Cosgrove, R Craik, MF da Silveira, D Danelon, T de Wet, E de Leon, S Deshmukh, G Deutsch, J Dhami, Nicola P Di, M Dighe, H Dolk, M Domingues, D Dongaonkar, D Enquobahrie, B Eskenazi, F Farhi, M Fernandes, D Finkton, S Fonseca, IO Frederick, M Frigerio, P Gaglioti, C Garza, G Gilli, P Gilli, M Giolito, F Giuliani, J Golding, MG Gravett, SH Gu, Y Guman, YP He, L Hoch, S Hussein, D Ibanez, C Ioannou, N Jacinta, N Jackson, YA Jaffer, S Jaiswal, JM Jimenez-Bustos, FR Juangco, L Juodvirsiene, M Katz, B Kemp, S Kennedy, M Ketkar, V Khedikar, M Kihara, J Kilonzo, C Kisiang'ani, J Kizidio, CL Knight, HE Knight, N Kunnawar, A Laister, A Lambert, A Langer, T Lephoto, A Leston, T Lewis, H Liu, S Lloyd, P Lumbiganon, S Macauley, E Maggiora, C Mahorkar, M Mainwaring, L Malgas, A Matijasevich, K McCormick, R McGready, R Miller, A Min, A Mitidieri, V Mkrtychyan, B Monyepote, D Mota, I Mulik, S Munim, D Muninzwa, N Musee, S Mwakio, H Mwangudzah, R Napolitano, CR Newton, V Ngami, JA Noble, S Norris, T Norris, F Nosten, K Oas, M Oberto, L Occhi, R Ochieng, EO Ohuma, E Olearo, I Olivera, MG Owende, C Pace, Y Pan, RY Pang, AT Papageorghiou, B Patel, V Paul, W Paulsene, F Puglia, M Purwar, V Rajan, A Raza, D Reade, J Rivera, DA Rocco, F Roseman, S Roseman, C Rossi, PM Rothwell, I Rovelli, K Saboo, R Salam, M Salim, L Salomon, Luna M Sanchez, J Sande, I Sarris, S Savini, IK Sclowitz, A Seale, J Shah, M Sharps, C Shembekar, YJ Shen, M Shorten, F Signorile, A Singh, S Sohoni, A Somani, TK Sorensen, A Soria- Frisch, E Staines Urias, A Stein, W Stones, V Taori, K Tayade, T Todros, R Uauy, A Varalda, M Venkataraman, C Victora, J Villar, S Vinayak, S Waller, L Walusuna, JH Wang, L Wang, S Wanyonyi, D Weatherall, S Wiladphaingern, A Wilkinson, D Wilson, MH Wu, QQ Wu, K Wulff, D Yellappan, Y Yuan, S Zaidi, G Zainab, JJ Zhang, Y Zhang

**Affiliations:** a Department of Physics, University of Wisconsin, Milwaukee, WI, USA; b Nuffield Department of Women's & Reproductive Health, University of Oxford, Oxford, UK; c Oxford Maternal & Perinatal Health Institute, Green Templeton College, University of Oxford, Oxford, UK; d Centre for Tropical Medicine and Global Health, Nuffield Department of Medicine, University of Oxford, Oxford, UK; e Department of Engineering Science, University of Oxford, Oxford, UK; f College of Health Sciences, University of Sharjah, University City, United Arab Emirates; g Centre for Global Child Health, Hospital for Sick Children, Toronto, ON, Canada; h Maternité Necker-Enfants Malades, Assistance publique – Hôpitaux de Paris (AP-HP), Université Paris Descartes, Paris, France; i Programa de Pós-Graduação em Epidemiologia, Universidade Federal de Pelotas, Pelotas, Brazil; j Programa de Pós-Graduação em Saúde e Comportamento, Universidade Católica de Pelotas, Pelotas, Brazil; k Faculty of Health Sciences, Aga Khan University, Nairobi, Kenya; l Department of Family & Community Health, Ministry of Health, Muscat, Oman; m Department of Obstetrics and Gynecology, University of Washington, Seattle, WA, USA; n Department of Global Health, University of Washington, Seattle, WA, USA; o Nagpur INTERGROWTH-21st Research Centre, Ketkar Hospital, Nagpur, India; p School of Public Health, Peking University, Beijing, China; q Shoklo Malaria Research Unit (SMRU), Mahidol-Oxford Tropical Medicine Research Unit (MORU), Faculty of Tropical Medicine, Mahidol University, Mae Sot, Thailand; r South African Medical Research Council Developmental Pathways for Health Research Unit, Department of Paediatrics & Child Health, University of the Witwatersrand, Johannesburg, South Africa; s Dipartimento di Scienze Pediatriche e dell' Adolescenza, Struttura Complessa Direzione Universitaria Neonatologia, Università di Torino, Torino, Italy; t Department of Obstetrics & Gynaecology, Division of Women & Child Health, Aga Khan University, Karachi, Pakistan; u Centre of Excellence in Women and Child Health, Aga Khan University, Karachi, Pakistan

## Abstract

**Background:**

Preterm birth is a major global health challenge, the leading cause of death in children under 5 years of age, and a key measure of a population's general health and nutritional status. Current clinical methods of estimating fetal gestational age are often inaccurate. For example, between 20 and 30 weeks of gestation, the width of the 95% prediction interval around the actual gestational age is estimated to be 18–36 days, even when the best ultrasound estimates are used. The aims of this study are to improve estimates of fetal gestational age and provide personalised predictions of future growth.

**Methods:**

Using ultrasound-derived, fetal biometric data, we developed a machine learning approach to accurately estimate gestational age. The accuracy of the method is determined by reference to exactly known facts pertaining to each fetus—specifically, intervals between ultrasound visits—rather than the date of the mother's last menstrual period. The data stem from a sample of healthy, well-nourished participants in a large, multicentre, population-based study, the International Fetal and Newborn Growth Consortium for the 21st Century (INTERGROWTH-21st). The generalisability of the algorithm is shown with data from a different and more heterogeneous population (INTERBIO-21st Fetal Study).

**Findings:**

In the context of two large datasets, we estimated gestational age between 20 and 30 weeks of gestation with 95% confidence to within 3 days, using measurements made in a 10-week window spanning the second and third trimesters. Fetal gestational age can thus be estimated in the 20–30 weeks gestational age window with a prediction interval 3–5 times better than with any previous algorithm. This will enable improved management of individual pregnancies. 6-week forecasts of the growth trajectory for a given fetus are accurate to within 7 days. This will help identify at-risk fetuses more accurately than currently possible. At population level, the higher accuracy is expected to improve fetal growth charts and population health assessments.

**Interpretation:**

Machine learning can circumvent long-standing limitations in determining fetal gestational age and future growth trajectory, without recourse to often inaccurately known information, such as the date of the mother's last menstrual period. Using this algorithm in clinical practice could facilitate the management of individual pregnancies and improve population-level health. Upon publication of this study, the algorithm for gestational age estimates will be provided for research purposes free of charge via a web portal.

**Funding:**

Bill & Melinda Gates Foundation, Office of Science (US Department of Energy), US National Science Foundation, and National Institute for Health Research Oxford Biomedical Research Centre.

## Introduction

The importance of accurately estimating fetal gestational age is widely known,^1^, ^2^, ^3^, ^4^, ^5^, ^6^ but Naegele's rule from 1812 is still used to estimate the likely duration of a pregnancy.^7^ The rule is also used in all estimates of fetal gestational age, if only to convert fetal biometric data to gestational age.

Naegele's rule rests on biologically questionable assumptions, including: the last menstrual period (LMP) is the appropriate time zero for pregnancy, and ovulation occurs on the 14th day of a 28-day menstrual cycle. In reality, the LMP is often unknown or poorly recalled, menstrual cycles can be irregular, and the time of ovulation may vary, even in women with regular menstrual cycles.^8^, ^9^, ^10^

Estimates of current gestational age represent moving averages over heterogeneous data recorded with substantial timing error. The inevitable scatter of individual data points about the average is regarded as noise. In all estimation techniques, this scatter increases—and the absolute accuracy of the gestational age estimate deteriorates—as the pregnancy advances.^3^, ^5^ For example, using ultrasound to measure the fetal head circumference mid-gestation to estimate gestational age assumes that all fetuses of the same gestation have the same measurement, which is intrinsically inaccurate. Consequently, accurate determination of gestational age, arguably one of the most important fetal characteristics, has remained challenging. WHO recommends ultrasound measurement of fetal size before 24 weeks of gestation to estimate gestational age, as current measures for estimating gestational age are particularly poor after 24 weeks of gestation, when many women, especially in low-resource settings, first present for pregnancy care. These inaccurate estimates are a major concern, because they affect estimates of preterm birth and small for gestational age rates in many settings, and because this issue in turn has important implications for the pregnancy care of individual women.^11^, ^12^

**Research in context**

**Evidence before this study**

We searched MEDLINE, Embase, Cochrane, and Web of Science with free-text terms and medical subject headings related to gestational age, ultrasound, fetal development, and second and third trimesters of pregnancy from Jan 1, 1970, to Dec 31, 2019. Reliable estimation of gestational age is essential for clinical care—particularly for the mother's antenatal care, assessment of fetal growth, accurate estimation of gestational age at birth, and to assess appropriateness of size at birth. Accurate estimates of gestational age are also essential at population level, specifically to calculate rates of preterm birth and small for gestational age, and for ongoing research into predicting pregnancy outcome, since biomarkers change with gestational age. Previous systematic reviews have shown that inaccurate estimations of gestational age mean measured rates are rough approximations to the truth, especially in geographical regions at greatest risk of preterm birth and small for gestational age. Ultrasound measurement of fetal crown rump length at 11–14 weeks is currently the most accurate method of gestational age estimation. However, in many settings women do not seek care in early pregnancy, and ultrasound dating in late pregnancy becomes necessary. Such measurements are even less accurate, because fetal growth charts have many methodological limitations, and fetal growth variations become more pronounced with time. All methods of ultrasound-based gestational age estimation have three fundamental problems. First, they ignore variations in the time of ovulation, which introduces substantial uncertainty in the start of pregnancy (time zero error). Second, they disregard the heterogeneity in fetal growth rates, seen even when the time of ovulation is accurately known. Third, they offer no guidance on the future growth trajectory of a given fetus, and hence no personalised indicator of potential risk. Between 20 and 30 weeks of gestation, the accuracy of even the best ultrasound estimates degrades steadily from 9 to 18 days. In the absence of alternatives, WHO recommends ultrasound measurement of fetal size before 24 weeks of gestation to estimate gestational age.

**Added value of this study**

In this study we use data from the prospective, multicentre, international, population-based project by the International Fetal and Newborn Growth Consortium for the 21st Century (INTERGROWTH-21st). Women received ultrasound scans every 5 weeks throughout pregnancy. The generalisability of the algorithm was tested with the INTERBIO-21st Fetal Study population. In this study population, data were collected in a fashion similar to INTERGROWTH-21st, but from women at higher risk of small for gestational age and preterm birth. Between 20 and 30 weeks of gestation, the gestational age estimates obtained with our new data-analytical approach are accurate to within 3 days. The algorithm also provides 6-week predictions of the growth trajectory of each fetus with an accuracy of 7 days. The accuracy of these estimates are verified by reference to exact, independently known facts about each fetus, specifically the dates of ultrasonographic measures.

**Implications of all the available evidence**

We developed a machine-learning approach, for which the uncertainty of gestational age estimation using ultrasound in the 20–30 weeks gestational age window is 3–5 times lower than estimates obtained with previous techniques. This has the potential to improve pregnancy care, facilitate public health measures, and substantially improve perinatal outcomes.

Although ultrasonography has made it possible to perform accurate fetal biometry, the measured dimensions must be converted to a gestational age, typically via LMP-based formulae or their derivatives.^13^ This gives rise to a fundamental problem: the accuracy of even the best watch can be no better than that of the fiducial master clock used to calibrate it. Not only are the clocks currently available to clinicians poorly synchronised (time zero error), each clock ticks at a different rate (fetal heterogeneity). Extrapolation of the best algebraic gestational age model^5^ to the start of pregnancy shows about a quarter of the total gestational age estimation error is due to the inexactly known time of conception, with the remaining three quarters stemming from differences in fetal growth rates (appendix pp 2–4).

Further progress requires an approach that is able to satisfy the following requirements. First, the method of gestational age estimation must substantially mitigate the effects of uncertainty about the time of conception and variations in fetal growth rates. Second, the accuracy of each gestational age estimate must be determined with reference to accurately known, easily accessible, fetus-specific, observable parameters. Third, the method must produce forecasts of future growth for each individual fetus (personal estimates and predictions), not population averages. Finally, the approach should help identify fetuses in need of closer monitoring.

In this study, we developed and tested a machine-learning approach to satisfy the requirements we outlined, and deliver highly accurate gestational age estimates and predictions of future growth. Machine learning, a branch of artificial intelligence, uses so-called training data to learn how best to capture the characteristics of a given type of data, in this case pertaining to fetal growth. Geometric machine learning, the technique used here, learns from the geometry of the data. A conceptual outline of the approach and the underlying mathematical details can be found in other studies,^14^, ^15^, ^16^ and in the appendix (pp 4–6, 8–12).

## Methods

### Algorithm development and validation

The accuracy of gestational age estimation algorithms is commonly determined by comparison with other estimation methods.^3^, ^5^, ^17^, ^18^ Because these methods rely directly or indirectly on Naegele's rule, this tends to propagate error, rather than quantify uncertainty. This problem can be circumvented by recourse to accurately known observables for each fetus. To establish the accuracy of our approach, we used three independent methods.

For method A, the algorithm is provided with two sets of ultrasound measures from a previously unseen (test) fetus and asked to determine the time interval separating them. No timing information is provided to the algorithm. Deviations from the accurately known time interval quantify the uncertainty in the information extracted from the data, including gestational age.

For method B, the algorithm is given a single set of previously unseen ultrasound measures obtained at one visit and asked to estimate gestational age. No timing information is provided to the algorithm. Gestational age estimates based on measures made during a single visit are possible in the majority of cases, because the estimate is often insensitive to the choice of the growth trajectory identified as characteristic of a specific fetus. The error in such estimates is defined as the discrepancy between the gestational age predicted from biometric measures made during one visit, and the gestational age estimated using measures from two visits, because the latter is deduced by comparison with the accurately known time elapsed between the two visits. In some cases, the gestational age estimate is sensitive to the choice of growth trajectory selected, causing the algorithm to return that “an estimate with accuracy better than the typical LMP-based estimates requires additional data”.

For method C, the algorithm is given fetal biometric measures from two visits without timing information and is asked to forecast the time of a subsequent scan of the fetus. Error is defined as the discrepancy between the forecast and the actual time of a subsequent visit.

### Data selection and sampling

To be useful, a machine-learning algorithm must be statistically accurate, and able to generalise from training data to previously unseen data, ideally from a different population. Using methods A, B, and C, we show the accuracy and generalisability of our approach with reference to data from two large, multicentre studies (appendix pp 16–18).

Dataset 1 pertains to 4607 healthy, well-nourished women with singleton pregnancies at low risk of adverse maternal and perinatal outcomes, who participated in the Fetal Growth Longitudinal Study (FGLS), one of the main components of the International Fetal and Newborn Growth Consortium for the 21st Century (INTERGROWTH-21st), a large, multicentre, longitudinal, population-based project conducted between 2009 and 2016, in eight delimited, diverse, geographical urban areas.^19^, ^20^

The data used for train and test of our algorithm were collected during the FGLS. Briefly, the study involved performing serial examinations with the same ultrasound machine (Philips HD9; Philips Healthcare, Andover, MA, USA) every 5 weeks (within 1 week either side) after an initial scan at less than 14 weeks of gestation that confirmed the certain LMP-based gestational age. Hence, the possible ranges of scan visits were at 14–18, 19–23, 24–28, 29–33, 34–38, and 39–42 weeks of gestation. The fetal anthropometric measures obtained at each visit after 14 weeks of gestation included head circumference , abdominal circumference, and femur length. Each parameter was measured in triplicate from three separately obtained ultrasound images of each structure. The measurement protocol (including masking of the ultrasonographer to the values) and the training, standardisation, and quality control procedures have been reported elsewhere.^19^, ^21^, ^22^, ^23^

The generalisability of the algorithm—ie, its ability to yield accurate estimates using fetal biometric measures from a different dataset (no part of which was used for training)—was established using dataset 2, from the INTERBIO-21st Study (phase 2 of the INTERGROWTH-21st Project).^24^ The protocol in the longitudinal component of INTERBIO-21st (the Fetal Study) was almost identical to that used in FGLS. However, the population was much more heterogeneous and women were at higher risk of small for gestational age and preterm birth, with the aim of improving the functional classification of preterm birth and fetal growth restriction.

**Figure 1:**
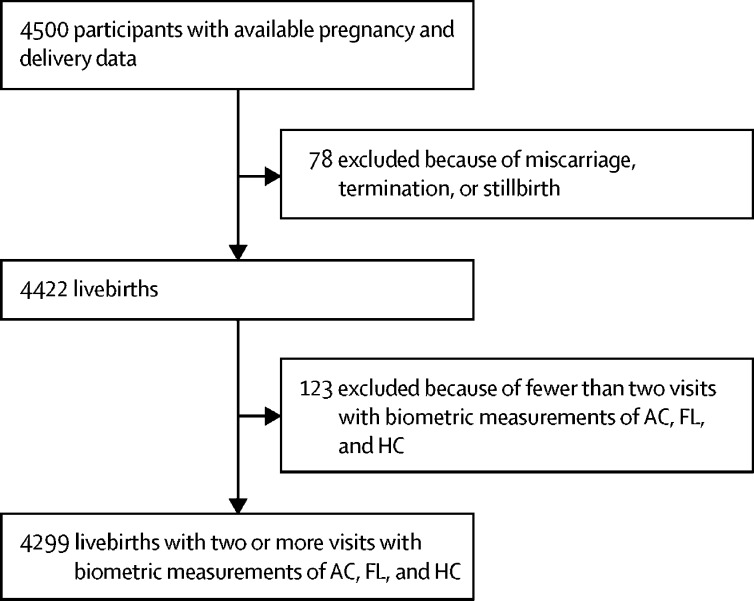
Flowchart used to select a subset of the participants in the INTERGROWTH-21st Fetal Growth Longitudinal Study for analysis The procedure closely follows that used by Papageorghiou and colleagues.^5^ INTERGROWTH-21st=International Fetal and Newborn Growth Consortium for the 21st Century. AC=abdominal circumference. FL=femur length. HC=head circumference.

The flowchart we used to select healthy FGLS participants for analysis (figure 1) is similar to that used by Papageorghiou and colleagues,^5^ thus allowing direct comparison of the results of previous analysis with the results obtained with the algorithm presented here. A total of 3076 participants in the INTERBIO-21st Fetal Study^24^ with complete data were included. In both datasets 1 and 2, the distribution of ultrasound data displays peaks at about monthly intervals. To prevent this non-uniform distribution from biasing our analyses, each train-and-test run was done on a randomly selected, uniform distribution of data. No participant was used for testing more than once in the study. We ensured that changing the number of analysed scans per day from 20 to 40 changed the 95% half-intervals by no more than 1 day. The most accurate results were obtained with 20 scans per day.

### Accuracy and generalisability assessments

The accuracy of our algorithm was assessed by a train-and-test approach with the FGLS dataset (dataset 1),^20^ using the analytical pipeline shown in the appendix (p 7). Briefly, participants were randomly divided into *N* subgroups. Each of the *N* subgroups was reserved in turn to serve later as the test data—ie, to measure the performance of the gestational age estimation algorithm with data not used in training. The participants in the other *N–*1 groups were pooled. Data vectors were randomly removed from each time bin to obtain a distribution of measures uniform in time. The resulting data were used for training. The performance of the algorithm was measured using the reserved test set. This train-and-test procedure was repeated until each of the *N* subgroups was used as the test dataset once, with the other *N*–1 subgroups used for training. The procedure resulted in *N* sets of test results, which were pooled to assess the statistical accuracy of the algorithm. The following values of *N* were used: 3, 4, 5, and 10. The 95% half-intervals obtained with different values of *N* differed by a fraction of 1 day. The results presented in this paper pertain to *N*=4, with 20 scans per day, but they were not sensitive to the choice of *N* over the range we have explored. To show generalisability, the algorithm produced by training with FGLS data^20^ was used to estimate gestational age using data from the INTERBIO-21st Fetal Study (dataset 2).^24^

The accuracy of our approach could be fully explored only over the period spanning 20 to 30 weeks of gestation, for two reasons. First, head circumference, abdominal circumference, and femur length data were available only after 14 weeks of gestation. This data truncation lead to reduced estimation accuracy before about 16 weeks of gestation. Second, our algorithm analyses a series of measures at a time.^15^ In the present study, each series consisted of 1024 measures. This reduced the total accessible timespan by about 8 weeks on each flank, which was further limited by the need for suitable measures within the truncated range. In principle, the accessible timespan can be extended by analysing shorter series of measures, or by using data more uniformly distributed in time, but the former can impose a noise penalty.

### Computational requirements

All statistical results presented here were obtained using MATLAB (release 2015b and 2019a). The training step, which needs to be done only once, can be accomplished in about 2 h on a Linux computer with a 12-core, 3GHz Intel Xeon CPU and 256 GB RAM. For field or clinical applications, the outcome of training can be pre-stored in software or hardware, requiring no more than a few megabytes of memory or storage. We plan to make the tool generally accessible for research purposes free of charge.

### Role of the funding source

The funders of the study had no role in study design, data collection, data analysis, data interpretation, or writing of the report. The corresponding author had full access to all the data in the study and had final responsibility for the decision to submit for publication.

## Results

**Figure 2:**
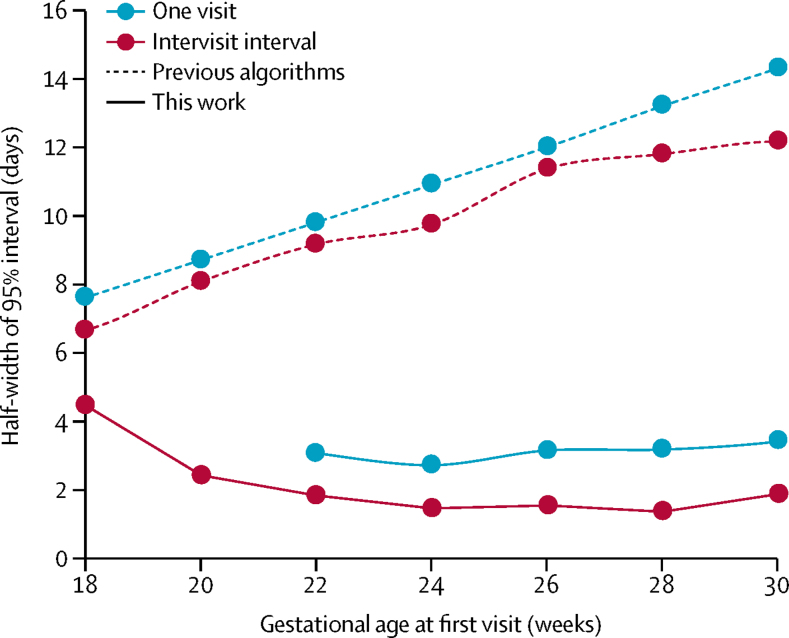
Algorithm accuracy in gestational age estimates based on single ultrasound visits or intervals between visits Accuracy of the new algorithm in estimating gestational age from ultrasound measurements of head circumference, abdominal circumference, and femur length. The Fetal Growth Longitudinal Study dataset^20^ of the International Fetal and Newborn Growth Consortium for the 21st Century was analysed. The uncertainty is expressed as the half-width of the 95% interval. For the solid red curve, the measure of error is the discrepancy between the algorithm's estimate of the time elapsed between two visits, and the actual time interval between the visits. The solid blue curve pertains to gestational age estimates based on a single set of biometric measurements. The error is the discrepancy between the algorithm's estimate and that obtained from two visits. For comparison, the reported error of a so-called genetic algorithm with the same data (but with mitigating strategies against truncation) by Papageorghiou and colleagues^5^ is shown in the dotted blue curve. The performance of the genetic algorithm is typical of the current state of the art. The dotted red curve shows the accuracy of the genetic algorithm when the intervisit interval is used as the measure of error. Using the intervisit interval as the measure of error modestly improves the estimation accuracy of current algorithms. This highlights the need to take fetal growth heterogeneity into account.

The accuracy of gestational age estimates obtained with FGLS data^20^ was measured by method A and method B (figure 2). Method A was based on the interval between two visits. Each data point represents the average over uncertainties resulting from intervisit intervals ranging from 4 to 10 weeks. The variation between results obtained from different intervals is ±1 day. For first scans between 20 and 30 weeks LMP-based gestational age, followed by a second scan 4 to 10 weeks later, the estimation error is less than 3 days. Fetal gestational age can thus be estimated in the 20–30 weeks gestational age window with a prediction interval 3–5 times better than with any previous algorithm. The error increases on both sides of the 20 to 30 weeks window of LMP-based gestational age. This is because of the reduction in time span imposed by data truncation and the need to consider concatenated series of measures, as outlined in the appendix (p 9). Method B was based on a single set of ultrasound measurements. This approach to estimating gestational age was applicable in the majority of cases with measurements between 22 and 30 weeks LMP-based gestational age.

We quantified the accuracy of our algorithm by reference to deviations from exactly known facts—specifically, intervisit intervals. We also assessed how other approaches perform, when their accuracy is measured against the exactly known interval between two visits. Figure 2 shows such an error estimation approach improves the accuracy of existing algorithms analysing the same data only modestly,^5^ with the errors remaining substantially larger than the algorithm presented in this paper. As detailed in the appendix (p 13), the error distribution in gestational age estimates obtained by our approach is extremely narrow, even outside the 95% uncertainty window.

**Figure 3:**
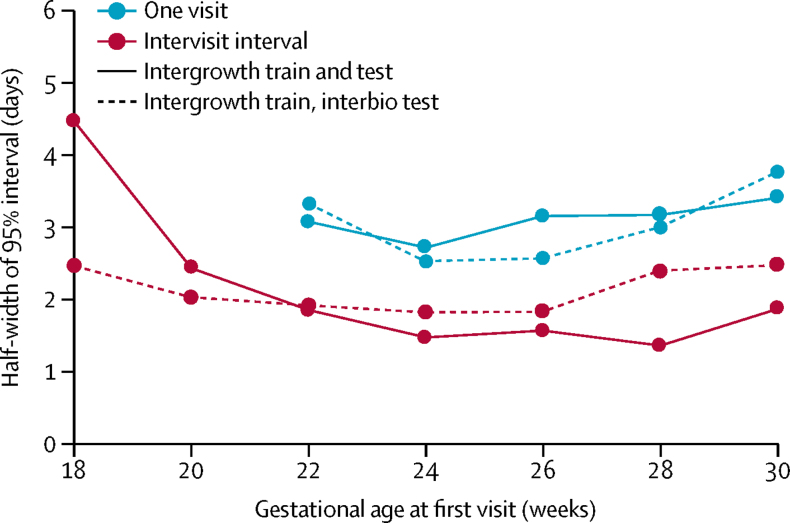
Accuracy of gestational age estimates obtained from different populations After training with subgroups of the Fetal Growth Longitudinal Study dataset^20^ of the International Fetal and Newborn Growth Consortium for the 21st Century, the algorithm was used to obtain gestational age estimates for members in different subgroups of the same population, as well as a members of a different population (INTERBIO-21st Fetal Study).^24^ Estimates obtained from intervisit intervals and single visits are both shown. Over the 20–30 gestational week window, the gestational age estimation uncertainties differ by at most 1 day.

We measured the generalisability of our approach to other datasets, specifically dataset 2, no part of which was used for training. Figure 3 shows there is no substantial degradation in the accuracy of gestational age estimation when the algorithm trained with data from one population^20^ is used to derive estimates for members of a different, far more heterogeneous population in dataset 2.^24^

**Figure 4:**
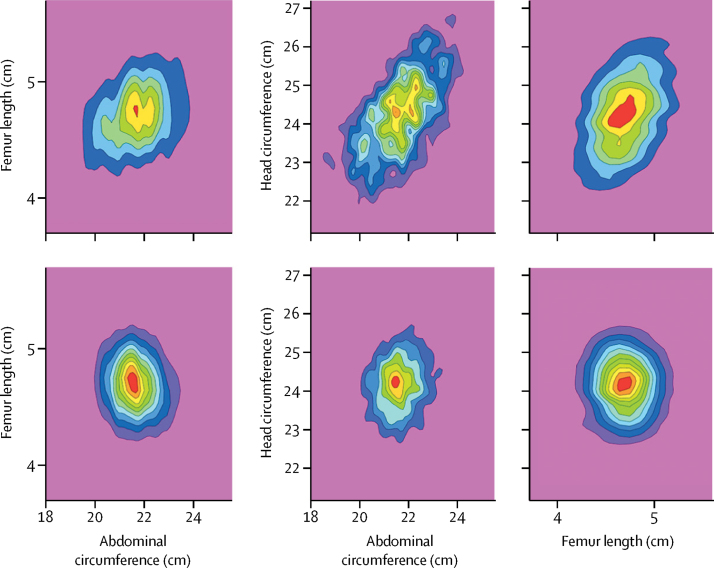
Probability distributions for fetal biometric variables at week 26 of pregnancy. The top row describes distributions compiled with standard estimates of gestational age.^5^ The complex, multipeaked character of the distributions are due to noise (uncertainty) in gestational age estimates obtained with standard techniques. The bottom row describes distributions compiled with gestational age estimates from the algorithm presented in this paper. The tighter, single-peaked distributions show the improvement in gestational age estimates, and would facilitate identification of fetal growth abnormalities.

As shown in figure 4, the complex and multipeaked nature of probability distributions for fetal biometric measures using standard estimates of gestational age are removed by the accurate estimates of gestational age obtained with our approach. This illustrates the potential effect of improved estimates of gestational age on fetal growth charts. Our algorithmic approach is also able to forecast the future growth trajectory for each fetus in method C, with an accuracy of 7 days for a 6-week forecast (appendix pp 10–11). The potential effect and appropriate use of this capability constitute future tasks.

## Discussion

In the context of two large datasets, we have estimated gestational age between 20 and 30 weeks of gestation with 95% confidence to within 3 days, a substantially better accuracy than what has been achieved so far.^5^ Knowledge of the gestational age of each pregnancy is crucial for good obstetric management, and a cornerstone of antenatal care. Indeed, some of the most effective evidence-based interventions are gestational-age dependent. Examples include induction of labor at term to reduce stillbirth,^25^ and giving antenatal corticosteroids to women at risk of early preterm birth.^26^ For this reason, routine pregnancy dating is recommended. However, due to the expense and limited availability of appropriate infrastructure, this is realised mainly in high-income settings. As menstrual dates can be inaccurate (or not recalled), the best method for gestational age estimation is ultrasound measurement of the fetal crown-rump length in the first trimester.^27^ However, this measurement also has limitations, as it assumes that all fetuses of the same gestation have the same measurement, and neglects biological variation. A circular argument is created, where fetal biometry is used for estimation of gestational age, which is then compared to another marker of fetal biometry later in pregnancy. Also, for women who do not attend the early pregnancy ultrasound—or where such a service is not available, especially in low-resource settings—gestational age is estimated later in pregnancy. Estimating gestational age later in pregnancy has a major limitation: using a simple translation of fetal biometry to gestational age makes it impossible to distinguish fetal growth aberrations from differences in gestational age. By estimating the change in gestation between two time points, our algorithm can be validated independently of gestational age—for example, if a fetus is scanned exactly 10 weeks apart, the gestational age between the two assessments has to change by 10 weeks. Departures from this interval can then be used to verify the accuracy of our approach.

It is reasonable to expect that the accurate estimates of gestational age made possible by the approach presented here would make a substantial contribution to improved clinical care at the individual level. At the population level, the much-improved accuracy of gestational age estimation would help improve the accuracy of reported preterm birth rates.^28^ Such improvements will be of particular benefit in low-resource settings, once inexpensive ultrasound devices are more widely available. The forecasts of future growth trajectories of individual fetuses can also help identify at-risk fetuses. The necessary computational facilities are modest and widely available in clinical settings. In principle, the approach could also be used with other multiparameter (vector) data, such as emerging techniques based on measuring cell-free RNA transcripts in maternal blood.^6^ Algorithms based on metabolic profiles have been used for gestational age estimation, based on postnatal cord and heel prick blood spots. These algorithms have been shown to estimate gestational age to within an average deviation of 1 week overall, but they have the disadvantage of becoming available only after the birth of the baby, meaning they are less useful for individual patient management during pregnancy.

Our approach has a number of strengths. These include the study design: a large, international, population-based project with prospective enrollment of women early in pregnancy, and longitudinal assessment throughout pregnancy. Detailed ultrasound protocols and quality control processes were in place, and measurements were obtained by masked operators, meaning that they were unable to view the resulting measures in real time to avoid expected-value bias. A novel algorithmic approach was used to develop a method of gestational age estimation, whose accuracy is based on exactly known facts pertaining to each fetus. The generalisability of this algorithm was shown with data from a different, more heterogeneous sample of women, obtained using otherwise almost identical study protocols. Although the method was validated in different datasets never encountered during development of the technique, prospective validation is needed, particularly to measure the sensitivity and specificity of the at-risk predictions. Naturally, validation with additional external datasets would further strengthen our conclusions.

The most substantial weakness of our approach at present concerns the limited time window of 20–30 weeks of gestation, which was imposed by the available data. This is mitigated by the enhanced estimation accuracy available to the large number of women seeking care during this time window, particularly in low-income and middle-income countries. One of the strengths of our study, namely the high quality of ultrasound measurements, could also be a weakness, as high-quality ultrasound measurements may not be available in some low-resource settings. However, poor quality of ultrasound would affect all current algorithms of gestational age estimation. Finally, it should be noted that the ideal scenario is not to use machine learning to estimate gestational age late in the second trimester or during the third trimester in pregnancy, but rather to organise health systems so that they can provide universal early prenatal visits. Nevertheless, the reality is that many women do not receive this level of care, so we believe our algorithm would contribute substantially to improving pregnancy care.

In conclusion, we have presented an algorithm able to estimate fetal gestational age from ultrasound measures with a 95% half-width of better than 3 days over a 10-week window in the second and third trimesters of pregnancy. The accuracy of all previous algorithms over the same time period ranges from 9–18 days.^3^ To our knowledge, our results represent the first time these levels of accuracy have been surpassed. 6-week forecasts of future growth of individual fetuses are also possible by our approach, with an accuracy of 7 days. No new instrumentation or computing facilities are needed. The general approach of our algorithm is likely to find applications in many settings, including those where accurate gestational age estimates can help save lives of countless babies at risk of preterm birth. For this purpose, the algorithm for gestational age estimates will be provided for research purposes free of charge, and ultimately via a web portal and mobile apps for use in remote settings.

## Data sharing

All documentation, protocols, data collection forms, and clinical tools are freely available on the INTERGROWTH-21st website. Software for gestational age estimation is available on GitHub.
