# Supplementary material for: Achieving accurate estimates of fetal gestational age and personalised predictions of fetal growth based on data from an international prospective cohort study: a population-based machine learning study
Source: Lancet Digit Health. 2020 Jun 23;2(7):e368–75. doi: 10.1016/S2589-7500(20)30131-X (PMC7323599; doi:10.1016/S2589-7500(20)30131-X)
Supplement: Supplementary appendix [file mmc1.pdf]

THE LANCET
Digital Health

Supplementary appendix

This appendix formed part of the original submission and has been peer reviewed. We post it as supplied by the authors.

Supplement to: Fung R, Villar J, Dashti A, et al. Achieving accurate estimates of fetal gestational age and personalised predictions of fetal growth based on data from an international prospective cohort study: a population-based machine learning study. *Lancet Digital Health* 2020; **2:** e368–75.

Supplementary appendix

Table of Contents

Section	Page No.
1. Uncertainty components in fetal GA estimates	2
2. Conceptual outline	4
3. Analytical pipeline	7
4. Geometric Machine Learning	8
5. Nonlinear Laplacian Spectral Analysis	9
6. Previously unseen fetuses	9
7. Forecasting fetal growth (Method C)	10
8. Data preprocessing & analysis	11
9. Comparative performance of GA estimation methods	13
10. Pseudo-code	14
11. Data and related acknowledgments	16
12. References	18

1. Uncertainty components in fetal GA estimates

The uncertainty in gestational age (GA) estimates can be separated into two components: a) the uncertainty in the time of conception; and, b) the uncertainty due to heterogeneity in fetal growth rates. We determine each component from the data in [1]. In the second and third trimesters, the total GA uncertainty is dominated by the component due to growth-rate heterogeneity.

Consider the uncertainty components:

a) Uncertainty in the time of conception ("time-zero") σ_0;
b) Heterogeneity in fetal growth rates σ_{het}.

As these components are independent, the total uncertainty is given by:

$$\sigma_T^2 = \sigma_0^2 + \sigma_{het}^2 \ . \tag{1}$$

The analysis below is based on the following premises:

1. The total uncertainty, σ_T, is a function of GA, defined as the mean GA of the population within the relevant time-bin (see Table 3 of [1]);
2. The time-zero component σ_0 is non-negative, and GA-independent;
3. The heterogeneity component σ_{het} depends on GA, is non-negative, and zero at GA=0.

Denoting GA by t for convenience, [1] offers two expressions for σ_T in days:

$$\sigma_T^{SD} = \left(6.492 \times 10^{-7}\right)t^3 + 2.991 \text{ , (Eq. (1) of [1]) ,} \tag{2}$$

$$\sigma_T^{SD} = 4.009 \times 10^{-2} t - 1.149 \text{ , (Eq. (2) of [1]) ,} \tag{3}$$

with the superscript *SD* indicating the standard deviation (rather than the 95% half-interval). Eq.(3) has a negative intercept at $t = 0$, i.e., a negative σ_0, and thus unphysical. By using a third-order polynomial of the form:

$$\sigma_T^{95\%} = at^3 + bt^2 + ct + d \tag{4}$$

to fit $\sigma_T^{95\%}$, we have verified that Eq.(2) satisfies the condition $\sigma_{het}(GA > 0) \geq 0$, i.e., the heterogeneity component is always non-negative.

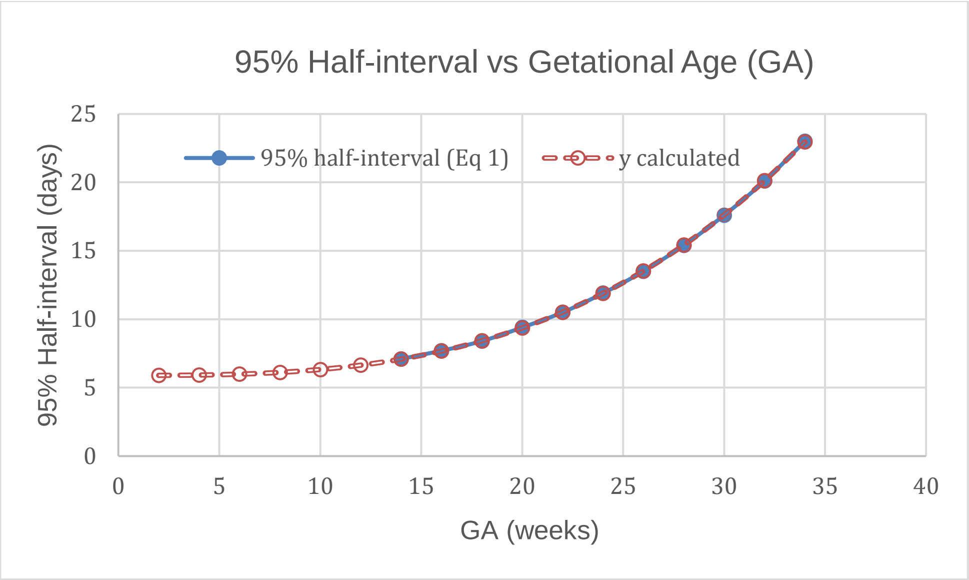

Figure. A1. Plot of total GA uncertainty vs. GA. The total uncertainty (blue) stems from [1]. Red pertains to Eq.(2) above.

The resulting time-zero uncertainty obtained from the $t = 0$ intercept of Eq.(2) is $\sigma_0^{95\%} = \sigma_T^{95\%}(t = 0) \simeq 6$ days. As shown in Fig. A2 below, 95% of the time-zero corrections performed by the geometric machine learning algorithm are contained within 7 days, in close agreement with the 6 days derived above. The (time-dependent) heterogeneity component can now be determined from:

$$\sigma_{het}(t) = \sqrt{(\sigma_T^2 - \sigma_0^2)} \ . \qquad (5)$$

The resulting 95% half-interval due to heterogeneity is shown in Fig. A3 below.

Implications for GA estimation

The time-zero contribution to the total uncertainty is at most 23% of the total uncertainty over the 20-30 weeks range, where the geometric algorithm currently operates. The 23% estimate is reached as follows. By definition, $\sigma_T(t = 0) = \sigma_0$. As previously stated, σ_0, the time-zero uncertainty is 6 days. From [1], at 20 weeks' GA, $\sigma_T = 9.4$ days, giving $\sigma_{het} = \sqrt{\left(9.4^2 - 6^2\right)} = 7.24$ days. Thus, $\frac{\sigma_{het}}{\sigma_T} = 0.77$. This means at 20 weeks' GA, leaving out the time-zero uncertainty changes the total uncertainty by 23%. At 30 week's GA, $\sigma_{het} = \sqrt{\left(17.6^2 - 6^2\right)} = 16.5 \quad \Rightarrow \frac{\sigma_{het}}{\sigma_T} = 0.94$, and leaving out the time-zero uncertainty changes the total uncertainty by only 6%.

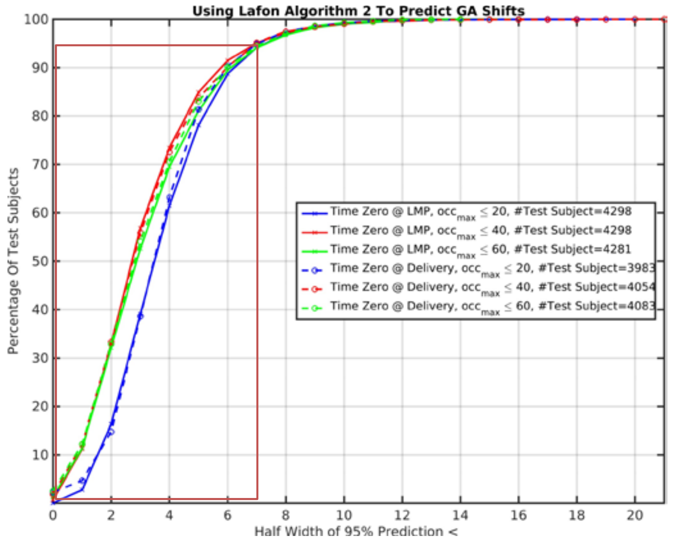

Figure A2. Plot of GA time-zero corrections performed by the geometric algorithm. The half-width of the time-zero correction covering 95% of "reads" for previously unseen ("test") data is 7 days.

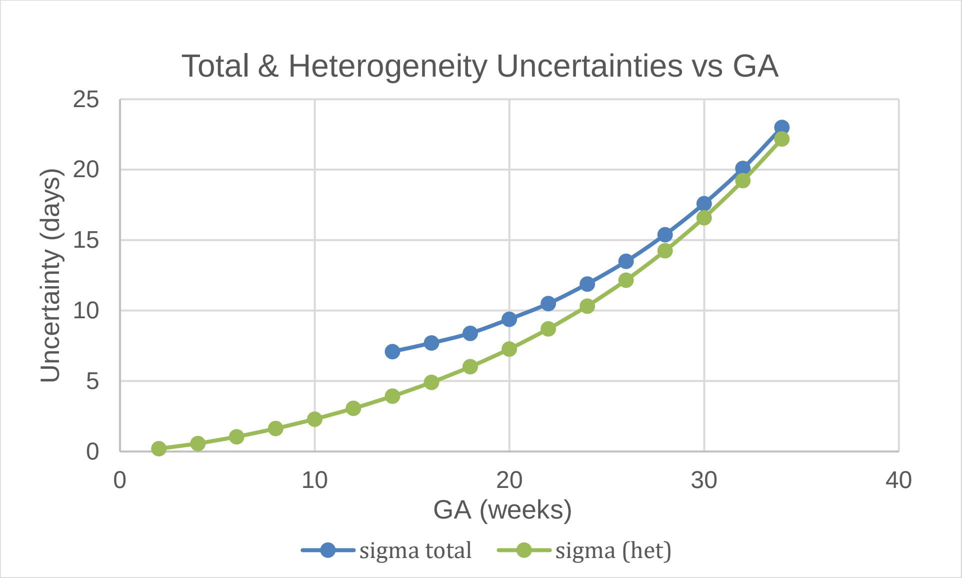

Figure A3. Total uncertainty in fetal GA estimates (blue), and the component due to heterogeneity in fetal growth rates (green).

2. Conceptual outline

Typically, an ultrasonographic fetal measure yields the values of a few biometric variables, e.g., the femur length, the head circumference, and the abdominal circumference. For simplicity, assume three biometric variables have been measured. Then an ultrasonographic measure can be represented as a point in three-dimensional space with each of *x*, *y*, and *z* corresponding to one of the three biometric variables

(Figure A4-A). A collection of such measures produces a cloud of points in three-dimensional space. In general, this cloud would itself be three-dimensional. But the three variables representing the fetal biometric dimensions are not independent. For example, a fetus with a long femur is likely to have a large head. This means the cloud of points formed by the collection of all fetal data has a dimensionality lower than three. Our analysis in fact shows the data from a large number of fetuses form a two-dimensional cloud. Technically, the cloud of points lie on a two-dimensional manifold, essentially a curved sheet. Identifying this manifold is helpful, because projecting the data points onto it filters out much of the noise inherent to real-world data.

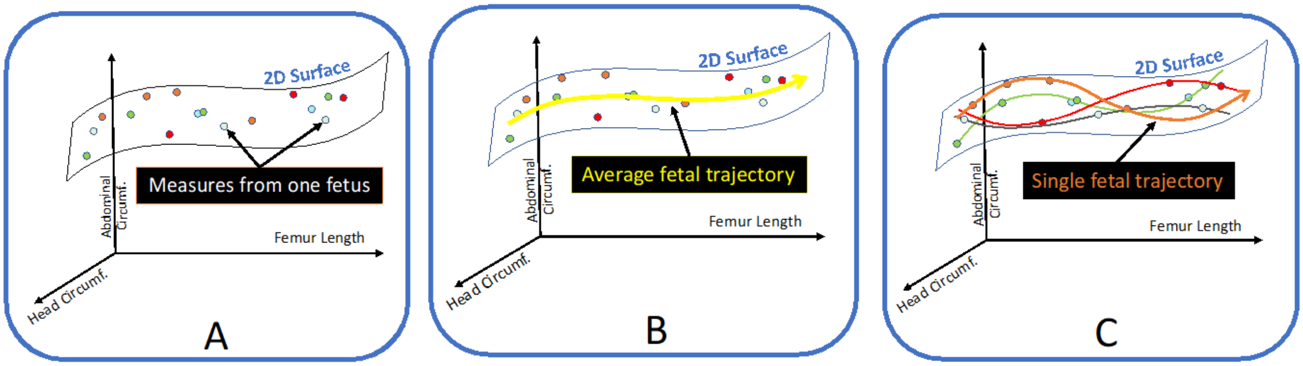

Figure A4. Schematic diagram of the approach used to determine the fetal gestational age and forecast its future growth.

Next, consider the data points obtained from a single fetus as the pregnancy advances (data points of the same color). Because the fetus is growing, these points define a line on the two-dimensional sheet. This line represents the growth trajectory of the fetus under consideration. One can deduce the growth trajectory of an "average" fetus by fitting a single line to all the data points on the manifold (Figure A4-B). This line represents the growth trajectory of a canonical ("model") fetus.

This fit stems from all fetuses in the dataset, each with a different (and inaccurately known) time of conception. The process of fitting a line in effect averages over these uncertainties, substantially reducing the uncertainty in the time of conception for the "model fetus" (yellow line of Figure A4-B).

This line is, nonetheless, highly informative; it encapsulates the properties of the class of lines, each of which represents the growth trajectory of a specific, yet to be identified fetus (Figure A4-C) (SA sections 4 - 7).

Given two or more sets of biometric data from a fetus, one can quickly identify the specific line best able to describe this fetus' growth trajectory. This is done by identifying the line best able to "predict" the time interval between successive biometric measures, which is accurately known. The discrepancy between this prediction and the known time interval between visits represents the error ("uncertainty") in our gestational

age estimation. In fact, for most fetuses, a single set of biometric data suffices to determine the fetal growth trajectory and estimate the gestational age with a prediction interval of less than 3 days.

Armed with the line best able to describe the growth trajectory of a given fetus, one can predict its future biometric data, and hence its future growth trajectory. Again, comparison with the time between actual measures allows us to estimate the prediction accuracy. The accuracy can be quantified by reference to the difference between predicted and actual fetal dimensions. More succinctly, it can be expressed as the uncertainty in a prediction of the fetal age, i.e., as the time-correction needed to eliminate the error in the fetal dimensions.

The conceptual outline presented above ignores important aspects of our approach. For example, simply fitting a line to all fetal data as implied above would obviate the possibility to extract fetus-specific information, much as averaging images of many people eliminates personal characteristics. As described in detail in [2] and in the SA sections 4 - 7, our machine-learning approach circumvents such problems. Broadly speaking, this type of capability is routinely demonstrated by increasingly ubiquitous facial-recognition technologies, which recognize individuals after training with populations of individuals. A discussion of these and other essential algorithmic features, described and experimentally validated elsewhere [2-4], is beyond the scope of the present paper.

3. Analytical pipeline

A schematic diagram of the analytical pipeline is shown in Fig. A5 below.

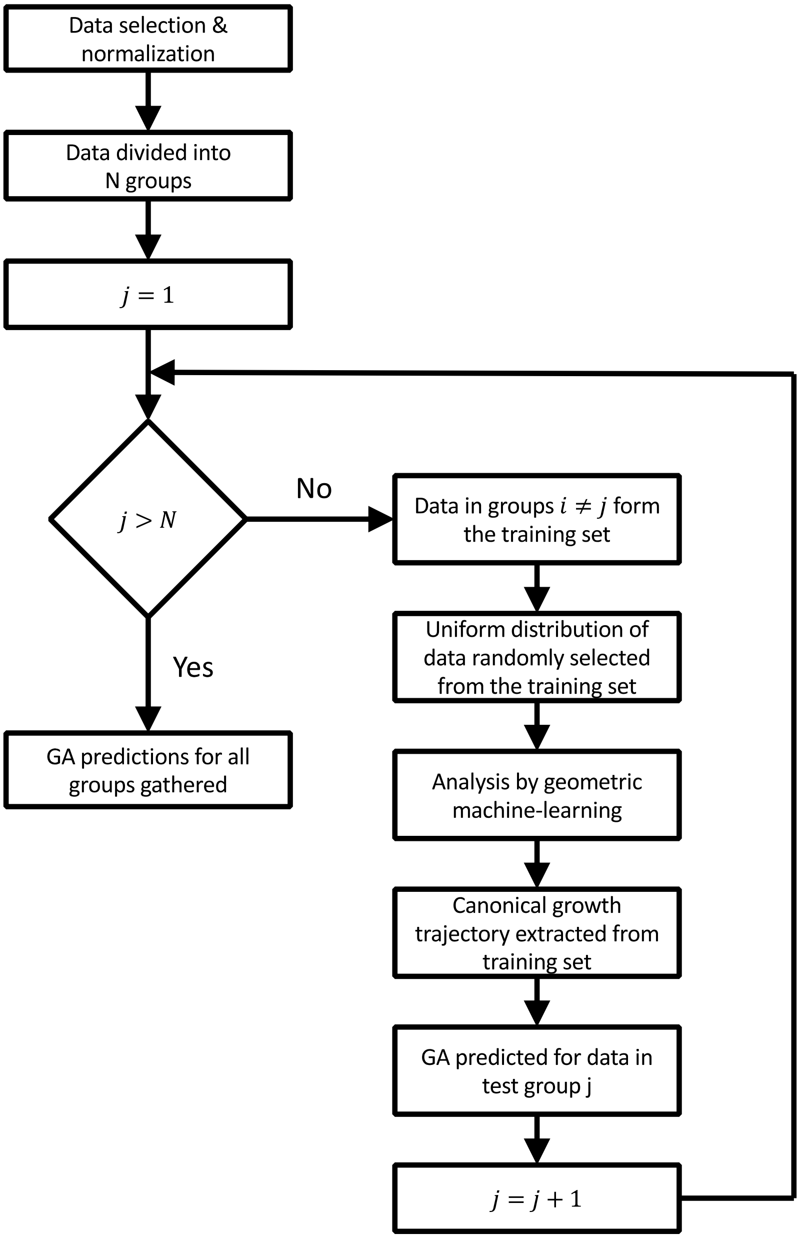

Figure A5. Schematic representation of the analytical pipeline.

4. Geometric Machine Learning

Geometric (manifold-based) Machine Learning is a key element of our analytical pipeline. This data-analytical approach is methodically transparent, mathematically rigorous, and unsupervised. (See, e.g., [2,5-7] and references therein.)

The algorithm accepts vector data as input. Here, each vector is comprised of an ultrasound measure of the fetal HC, AC, and FL time-stamped according to a LMP-based estimate of the gestational age (GA_{LMP}) [1].

A fetal biometric measure can be represented as a point in the three-dimensional space spanned by HC, AC and FL. Taken together, the data produce a cloud of points in this space, with each point representing a data vector. It is, however, more meaningful to resort to a space spanned by functions, more specifically eigenfunctions of the Laplace-Beltrami operator, which are learned from the data to reflect the intrinsic geometry of the dataset.[8] In this more abstract representation, the data cloud defines a curved hyperplane – a manifold. The distance between data points on this manifold is a measure of their similarity, with shorter distances representing closer similarity. At this stage, no timing information is used.

In this picture, the manifold represents all measures made available to the algorithm, with the developmental trajectory of a particular fetus corresponding to a specific trajectory on the manifold. This trajectory connects a time-ordered series of measures from a fetus (Fig. A6). As shown in Fig. A5 and outlined in Methods section of the paper, we use a portion of the data to obtain the manifold (“training”), with the remainder of the data reserved to evaluate performance (“test”).

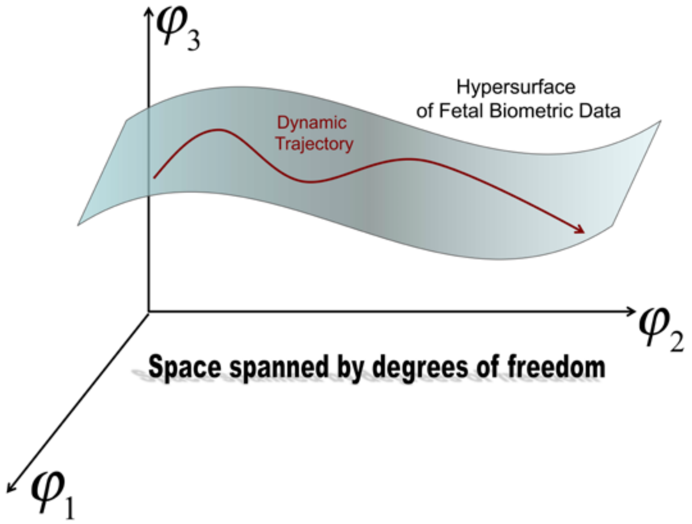

Figure A6. Schematic diagram of the data manifold formed by the dataset, and a particular growth (dynamic) trajectory on the manifold.

5. Nonlinear Laplacian Spectral Analysis

As shown in detail elsewhere [2], timing jitter and measurement noise can be substantially reduced by singular-value analysis of "supervectors" on the manifold by Nonlinear Laplacian Spectral Analysis (NLSA) [7]. Each supervector consists of a concatenated set of 1024 data vectors ordered according to the available (i.e., inaccurate) timestamps. The outcome of the NLSA approach is the jitter-corrected, noise-reduced dynamical trajectory of the data used for training [2]. The approach also reveals the characteristic combinations of measures acting as principal components of fetal growth, ranked in order of power (see Fig. A7).

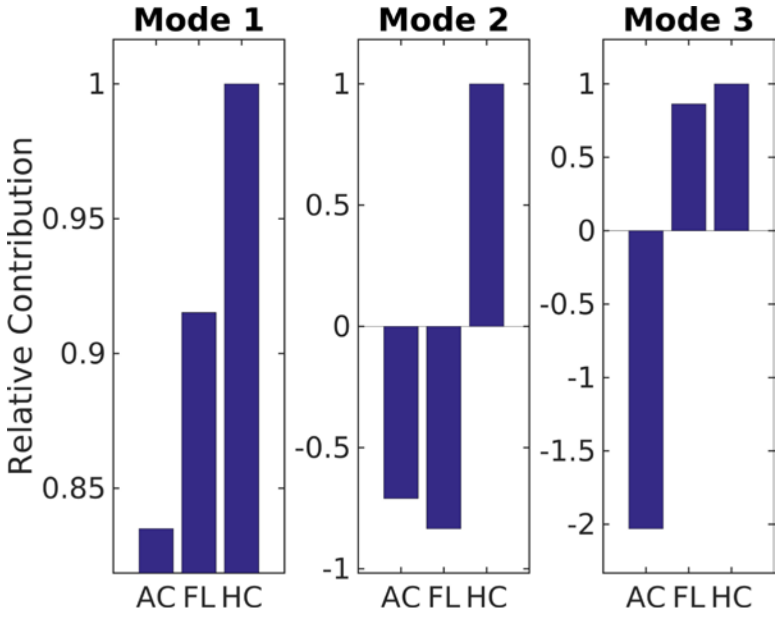

Figure A7. The top three characteristic modes determining fetal growth in our analysis. As the relative contribution of each measure is time-dependent, time-averages are shown. The relative amplitudes of the three modes are 1 : 0.04 : 0.01.

6. Previously unseen fetuses

GA estimation

Predicting the GA of a previously unseen fetus proceeds as follow. The eigenfunctions obtained by training are confined to the manifold. These manifold eigenfunctions must be extended to previously unseen data. We use the Nyström extension scheme outlined in [9], and described in more detail below. This approach self-consistently varies the width of a kernel (here denoted σ) to extend the manifold eigenfunctions to previously unseen data, subject to user-specified bounds on the power lost due to truncating eigenfunctions beyond a certain number k. We use this scheme, with minor modifications, to generate a family of extended eigenfunctions, each characterized by a different set of σ and k. For a particular, previously unseen fetus, we select that member of the family, which best predicts the independently known time interval between two ultrasound scans. With the appropriate extended eigenfunctions in hand, the GA can be estimated for each fetus (Method A in main text).

Accuracy of GA estimates

We infer the error in our estimates by reference to the discrepancy between the predicted and actual time intervals between the ultrasound measures. The accuracy of better than 3 days (95% half-interval) is maintained for first visits between 20 and 30 weeks' gestation followed by a second visit within 10 weeks of the first visit. This corresponds to a total time span of 40 weeks, including the 10-week intervisit gap. These results quantify the ability of our approach to recover reliable dynamical information over timespans comparable with a term pregnancy. Our error estimates are validated by a number of different train/test data splits (Fig. A5).

7. Forecasting fetal growth (Method C)

As outlined above, our approach identifies the class of growth trajectories best able to describe fetal growth dynamics. These so-called empirical functions are derived from the training data, which cover the entire pregnancy. One can, therefore, use these functions to forecast the future growth trajectory of any previously unseen fetus. Specifically, having selected the function best able to reproduce an intervisit interval for a specific fetus, the algorithm can be asked to use the same functions to predict the intervals to subsequent measurements for the same fetus. These predictions can then be compared with the actual known intervals.

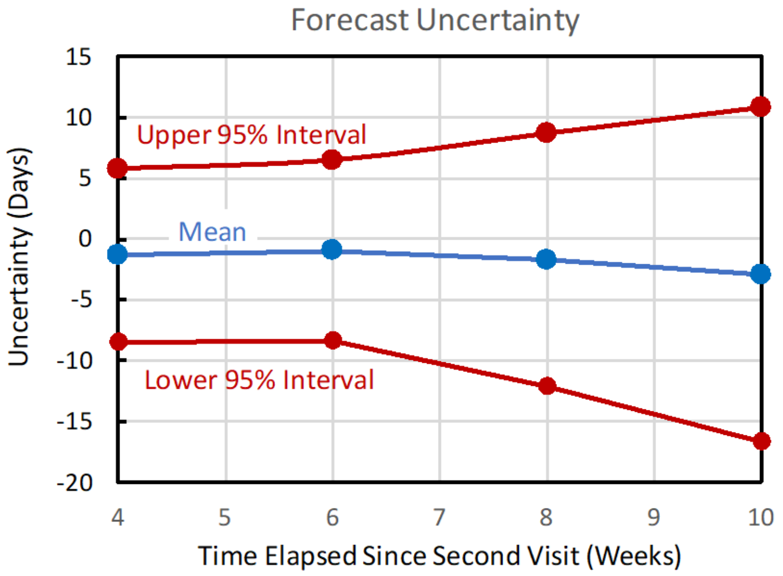

Figure A8. Accuracy of forecasting fetal growth.

As shown in Figure A8, the error in such forecasts consists of two components: a systematic shift of the mean (bias); and dispersion about the mean. As bias is the average over an ensemble, it can be determined in training and subtracted. After correcting for the bias, the 6-week forecast uncertainty is 7 days. With the current data, we are able to demonstrate this capability only when the second visit occurs between 22 and 24 weeks of gestation. As forecasts are necessarily less accurate than present-day measures, the 7-

day error estimate of *Method C* strongly corroborates the estimates obtained by *Methods A* and *B*.

8. Data preprocessing & analysis

The dataset used here consists of 4299 participants, and 20870 measurements of HC, AC and FL each at a GA estimated from GA_{LMP}. Each biometric variable was normalized through division by the largest value of the variable in the dataset, to place all three variables on the same footing. To ensure the analysis is not biased by variations in the number of snapshots per time interval, the distribution of measures in time was rendered uniform by random subsampling of the training data. Following a procedure described and validated elsewhere [10], training data were ordered according to their inaccurate timestamps and concatenated to form supervectors, each consisting of 1024 frames.

The manifold of supervectors was obtained at the optimum manifold-embedding kernel, as determined previously [11]. The supervectors were subjected to NLSA to mitigate timing jitter. NLSA is, in essence, a singular-value decomposition [12] on the curved manifold of supervectors, taking into account the Riemannian measure [7].

Unless otherwise stated, the results presented in this paper are robust to changes in parametric values, including the kernel width, the number of nearest neighbors and the concatenation parameter. Further details of the approach and its robustness to parametric choices are available in [10].

This analysis yields a canonical description of fetal growth, essentially free of timing jitter. The outcome of the algorithm consists of $(N - 2c + 1)$ frames of a movie, with N the initial number of single frames, and c the number of frames in each superframe. Each output frame constitutes a snapshot of the canonical fetal dimensions at an accurately known time point.

For the training data, the GA can be expressed as a function on the learned manifold:

$$GA(x) = \sum_{\ell \geq 0} c_{\sigma,\ell} \varphi_{\sigma,\ell}(x) ,$$

where the index x refers to a frame of the canonical development trajectory (a triplet of biometric measures), and σ the width of the kernel used in the expansion.

This expansion can be extended to previously unseen data by a Nyström extension scheme described in [9]. The scheme can be used to generate a family of eigenfunctions denoted by the width of the kernel $\boldsymbol{\sigma}$.

The GA of a previously unseen fetus can now be expanded in terms of the extended eigenfunctions $\bar{\varphi}_{\sigma,\ell}(\bar{x})$:

$$\overline{GA}_{\sigma,k}(\bar{x}) \equiv \sum_{\ell < k} c_{\sigma,\ell} \bar{\varphi}_{\sigma,\ell}(\bar{x}) .$$

The extension involves members of a family denoted by two parameters: the number of extended eigenfunctions k used in the sum above, and the kernel width σ.

The time interval between two visits is exactly known for each fetus. This information can be used to identify the (σ, k), which minimizes the discrepancy between the predicted and known intervisit time intervals (Method A in main text). The parameters σ and k constitute fetus-specific, i.e., “personal”, parameters. In practice, one selects the appropriate (σ, k) from a prestored databank.

The recorded GA for individual visits suffers from time-zero uncertainty, but the intervisit time interval - the time *difference* between the visits of a given participant - is immune to shifts in time-zero. Choosing (σ, k) based on the intervisit interval thus allows one to identify the set of extended functions best able to describe the fetal growth dynamics, independently of time-zero uncertainty. Thus, the approach described above can be used to determine the GA and growth trajectory of each previously unseen fetus, without being compromised by time-zero uncertainty.

For the majority of the participants, our approach can provide an accurate GA estimate from a single set of ultrasound measures (i.e., a single visit) (Method B in main text). Such cases are characterized by insensitivity to the choice of (σ, k), which results in a strongly peaked histogram of GA predictions. For the remaining cases, the algorithm returns that “an estimate more accurate than that based on LMP requires a further ultrasound examination after a suitable time interval”; or “a different approach for gestational age estimation may be needed”.

9. Comparative performance of GA estimation methods

	Current Clinical Methods[1]		New Method (Single Visit)		New Method (Two Visits)	
Biometric Measures	HC	HC & FL	HC, FL, & AC			
GA (Weeks)	Half-width of 95% prediction interval	Half-width of 95% prediction interval	Half-width of 95% prediction interval	Percentage of cases where estimation is possible*	Half-width of 95% prediction interval	Percentage of cases where estimation is possible
20	9.4	8.7	N/A	N/A	2.4	100%
22	10.5	9.8	3.1	22.4%	1.8	100%
24	11.9	10.9	2.7	61.2%	1.5	100%
26	13.5	12.0	3.1	71.2%	1.6	100%
28	15.4	13.2	3.2	66.6%	1.4	100%
30	17.6	14.3	3.4	37.0%	1.9	100%

Table. A1. GA estimation performance of current clinical methods[1] and the new machine learning algorithm. All 95% prediction intervals are in days. Results presented here pertain to a particular train/test run (dataset divided into 4 groups, 20 visits per day-bin used in training), but results for different train/test runs vary by no more than a day in prediction interval, and/or a few percentage points in the number of single-visit estimates.

* Single-visit cases yielding no estimate in a particular train/test run can produce an estimate in a different run (e.g., with a different number of visits per day in training). By combining results of multiple runs, GA estimates can be produced for practically all single-visit data in the 22-30 week window.

	New Method (Single Visit)		New Method (Two Visits)	
GA (Weeks)	Percentage with estimation error > 1 week	Percentage with estimation error > 2 weeks	Percentage with estimation error > 1 week	Percentage with estimation error > 2 weeks
20	N/A	N/A	0.7%	0.0%
22	0.0%	0.0%	0.4%	0.0%
24	0.0%	0.0%	0.4%	0.0%
26	0.7%	0.1%	0.3%	0.0%
28	0.4%	0.1%	0.2%	0.0%
30	0.5%	0.0%	0.4%	0.1%

Table. A2. Distribution of the GA estimation error for new estimation method.

10. Pseudo-code

The following pseudo-code clarifies the structure and implementation of the algorithm.

Training

Input

Ultrasound measurements.
Recorded GA at times of visits.

Output

Model data $\{x\}$, at uniformly spaced GA time points $\{GA(x)\}$.
A family of Diffusion Map eigenfunctions $\{\varphi_{\sigma,\ell}(x)\}$ for the model data.
The set of $\{c_{\sigma,\ell}\}$ coefficients.

Steps

i) Training data selected to give a uniform GA histogram.
ii) NLSA reconstruction of training data yields canonical developmental trajectory: model data $\{x\}$, at uniformly spaced GA time points $\{GA(x)\}$.
iii) Embed model data with Diffusion Map at various kernel widths to create a family of eigenfunctions $\{\varphi_{\sigma,\ell}(x)\}$ of the Laplace-Beltrami operator.
iv) For each kernel width σ, do.
v) Obtain the set of $\{c_{\sigma,\ell}\}$ coefficients by inverting $GA(x) = \sum_{\ell\geq 0} c_{\sigma,\ell}\varphi_{\sigma,\ell}(x)$.
vi) endfor.

Reading (More than one visit of the same subject)

Input

Ultrasound measurements for each visit in a vector $\bar{x}$.
Time interval(s) between visits.

Output

Predicted GA.

Steps

i) For each visit, do.
ii) For each member of the family of Diffusion Map eigenfunctions, and the associated kernel width σ, do.
iii) Nystrom extension yields the extended eigencomponents $\{\bar{\varphi}_{\sigma,\ell}(\bar{x})\}$.
iv) For different number k of Diffusion Map eigenfunctions, do.
v) Predicted GA for current (σ, k) is $\overline{GA}_{\sigma,k}(\bar{x}) \equiv \sum_{\ell<k} c_{\sigma,\ell}\bar{\varphi}_{\sigma,\ell}(\bar{x})$.
vi) endfor.
vii) endfor.
viii) endfor.

ix) For each member of the family of Diffusion Map eigenfunctions, and the associated kernel width σ, do.
x) For different number k of Diffusion Map eigenfunctions, do.
xi) Calculate intervisit time interval(s) from predicted GA's for current (σ, k).
xii) Calculate error(s) in intervisit time interval(s).
xiii) endfor.
xiv) endfor.
xv) Return GA's for (σ, k) with smallest intervisit time interval error(s).

Reading (One visit)

Input

Ultrasound measurements for one visit in a vector $\bar{x}$.

Output

Predicted GA, or message "An estimate with accuracy better than the typical LMP-based estimates requires additional data".

Steps

i) For each member of the family of Diffusion Map eigenfunctions, and the associated kernel width σ, do.
ii) Nystrom extension yields the extended eigencomponents $\{\bar{\varphi}_{\sigma,\ell}(\bar{x})\}$.
iii) For different number k of Diffusion Map eigenfunctions, do.
iv) Predicted GA for current (σ, k) is $\overline{GA}_{\sigma,k}(\bar{x}) \equiv \sum_{\ell<k} c_{\sigma,\ell}\bar{\varphi}_{\sigma,\ell}(\bar{x})$.
v) endfor.
vi) endfor.
vii) Make a histogram of all the predicted GA.
viii) If histogram peak exists.
ix) Return location of histogram peak as predicted GA.
x) else.
xi) Display message "An estimate with accuracy better than the typical LMP-based estimates requires additional data".
xii) endif.

Nystrom Extension

Input

Vectors $\{x_j\}$.
Kernel width σ.
Diffusion Map eigenvalues/ eigenfunctions $\{\lambda_\ell, \varphi_\ell\}$ for vectors $\{x_j\}$ with kernel width σ.
Vector $\bar{x}$.

Output

Extended eigencomponents $\{\bar{\varphi}_\ell\}$ for vector $\bar{x}$.

Steps

i) For each vector in $\{x_j\}$, do.

ii) Calculate squared Euclidean distance d_j^2 between vector x_j and vector $\bar{x}$.

iii) Calculate kernel $K_j = exp\left(-d_j^2/\sigma^2\right)$.

iv) endfor.

v) Normalize kernel to give $\{K_j^{norm}\}$.

vi) For each eigenvalue/ eigenfunction $\{\lambda_\ell, \varphi_\ell\}$, do.

vii) Calculate extended eigencomponent $\bar{\varphi}_\ell = \frac{1}{\lambda_\ell}\sum_j K_j^{norm}\varphi_\ell(x_j)$.

viii) endfor.

ix) Return extended eigencomponents $\{\bar{\varphi}_\ell\}$.

NLSA (time series)

Input

Data snapshots $\{x\}$ (possibly noisy) at given time points (possibly nonuniformly-spaced).

Timestamps $\{t\}$ of snapshots.

Output

Data snapshots $\{\bar{x}\}$ (noise reduced) at uniformly-spaced time points.

Timestamps $\{\bar{t}\}$ of snapshots.

Steps

i) Order snapshots $\{x\}$ based on given timestamps $\{t\}$.

ii) Concatenate ordered snapshots to give superframes X.

iii) Concatenate and average ordered timestamps to give timestamps of superframes.

iv) Embed superframes with Diffusion Map to obtain eigenfunctions φ of the Laplace-Beltrami operator and Riemannian measure μ.

v) Project superframes onto the embedding space: $A = X\mu\varphi$.

vi) Perform singular-value decomposition of A and retain only singular modes with significant singular values: $A = USV^T$, $U \xrightarrow[high\,S]{} \bar{U}$, $V \xrightarrow[high\,S]{} \bar{V}$, $S \xrightarrow[high\,S]{} \bar{S}$.

vii) Back-project: $\bar{X} = \bar{U}\bar{S}\bar{V}^T\varphi^T$.

viii) Unwrap $\bar{X}$ to give data snapshots $\{\bar{x}\}$.

ix) Concatenate and average superframe timestamps to give $\{\bar{t}\}$.

x) Return data snapshots $\{\bar{x}\}$ and timestamps $\{\bar{t}\}$.

11. Data and related acknowledgments

The INTERGROWTH-21st Project was approved by the Oxfordshire Research Ethics Committee “C” (reference: 08/H0606/139), and the research ethics committees of the individual institutions and the regional health authorities where the project was implemented. Written informed consent was obtained from all participants. The sponsors

had no role in the study design, data collection, analysis, interpretation of the data, or writing of the paper. The following authors had access to the full raw dataset: RF, JV, SHK, ATP and AO. The corresponding author had full access to all the data and final responsibility for submitting the paper.

Our first dataset pertained to 4607 healthy women with singleton pregnancies at low risk of adverse maternal and perinatal outcomes, who participated in the *Fetal Growth Longitudinal Study (FGLS),* one of the main components of INTERGROWTH-21st Project, was a large, multicenter, longitudinal, population-based project conducted between 2009 and 2016, in eight delimited diverse geographical urban areas: Pelotas (Brazil), Turin (Italy), Muscat (Oman), Oxford (UK), Seattle (USA), Shunyi County in Beijing (China), the central area of Nagpur (India), and the Parklands suburb of Nairobi (Kenya) [13,14]. The primary aim was to study growth, health, nutrition, and neurodevelopment from early pregnancy to 2 years of age in populations of optimally healthy mothers. A geographical area was a complete city or county, or part of a city with clear political or geographical limits, located at an altitude <1600m, with low-risk health indicators for perinatal morbidity and mortality, in which women receiving antenatal care had plans to give birth within the area free of, or with low levels of major, known, non-microbiological contamination [13].

In the FGLS, pregnant women were recruited from the aforementioned populations, if they met the individual entry criteria of health, nutrition, education, and socioeconomic position, and accurate gestational age estimation based upon certain LMP, regular menstrual cycles and ultrasound confirmation of gestational age in the first trimester. The objective was to construct international standards for gestational weight gain, early and late fetal growth, newborn size and preterm postnatal growth [15-19]. The cohort enrolled in FGLS was followed up to 2 years of age, and evaluated for skeletal growth, nutrition, health, and the WHO gross motor milestones, as well as neurodevelopment and associated behaviors [20-22].

The second dataset pertained to an unselected cohort of women recruited from six geographically diverse settings as part of the *INTERBIO-21st Fetal Study*, another main component of the INTERGROWTH-21st Project between February 2012 and December 2015 (our second dataset). The first three – Pelotas (Brazil), the Parklands suburb of Nairobi (Kenya), and Oxford UK – had been FGLS sites, whilst the others – Karachi (Pakistan), Mae Sot (Thailand), and Soweto (South Africa) – were chosen to include in the cohort with women at higher risk of pregnancy complications and adverse perinatal outcomes because of exposures, such as malnutrition, malaria and HIV. All the women initiated antenatal care before 14 weeks of gestation; gestational age estimation was based on measurement of fetal Crown-Rump Length as this higher risk population was expected to have less regular LMP. All women underwent serial examinations with the same ultrasound protocol as FGLS, every 5 weeks (within 1 week either side) after an initial scan <14 weeks of gestation, and the growth and neurodevelopment of their infants were assessed at 2 years of age.

We thank the Health Authorities in Pelotas, Brazil; Beijing, China; Nagpur, India; Turin, Italy; Nairobi, Kenya; Muscat, Oman; Karachi, Pakistan; Soweto, South Africa; Mae Sot, Thailand; Oxford, UK and Seattle, USA, who facilitated the project by allowing participation of these study sites as collaborating centers. We are grateful to Philips Medical Systems, who provided the ultrasound equipment and technical assistance throughout the project. We thank MedSciNet U.K. Ltd for setting up the INTERGROWTH-21st website and for the development, maintenance and support of the online data management system.

Finally, we thank the parents and infants who participated in the studies and the more than 200 members of the research teams who made the implementation of this project possible. The participating hospitals included: Brazil, Pelotas (Hospital Miguel Piltcher, Hospital São Francisco de Paula, Santa Casa de Misericórdia de Pelotas, and Hospital Escola da Universidade Federal de Pelotas); China, Beijing (Beijing Obstetrics & Gynecology Hospital, Shunyi Maternal & Child Health Centre, and Shunyi General Hospital); India, Nagpur (Ketkar Hospital, Avanti Institute of Cardiology Private Limited, Avantika Hospital, Gurukrupa Maternity Hospital, Mulik Hospital & Research Centre, Nandlok Hospital, Om Women's Hospital, Renuka Hospital & Maternity Home, Saboo Hospital, Brajmonhan Taori Memorial Hospital, and Somani Nursing Home); Kenya, Nairobi (Aga Khan University Hospital, MP Shah Hospital and Avenue Hospital); Italy, Turin (Ospedale Infantile Regina Margherita Sant' Anna and Azienda Ospedaliera Ordine Mauriziano); Oman, Muscat (Khoula Hospital, Royal Hospital, Wattayah Obstetrics & Gynaecology Poly Clinic, Wattayah Health Centre, Ruwi Health Centre, Al-Ghoubra Health Centre and Al-Khuwair Health Centre); Pakistan (Aga Khan University Hospital, Karachi); South Africa (Baragwanath Hospital, Soweto); Thailand (Shoklo Malaria Research Unit, Mae Sot); UK, Oxford (John Radcliffe Hospital) and USA, Seattle (University of Washington Hospital, Swedish Hospital, and Providence Everett Hospital).

12. References

1. Papageorghiou AT, Kemp B, Stones W, et al. Ultrasound-based gestational-age estimation in late pregnancy. *Ultrasound Obstet Gynecol* 2016; **48**(6): 719-26.
2. Fung R, Hanna AM, Vendrell O, et al. Dynamics from noisy data with extreme timing uncertainty. *Nature* 2016; **532**(7600): 471-5.
3. Wosiak A, Zamecznik A, Niewiadomska-Jarosik K. Supervised and Unsupervised Machine Learning for Improved Identification of Intrauterine Growth Restriction Types. *Proceedings of the 2016 Federated Conference on Computer Science and Information Systems (Fedcsis)* 2016; **8**: 323-9.
4. Naimi AI, Platt RW, Larkin JC. Machine Learning for Fetal Growth Prediction. *Epidemiology* 2018; **29**(2): 290-8.
5. Coifman RR, Lafon S, Lee AB, et al. Geometric diffusions as a tool for harmonic analysis and structure definition of data: diffusion maps. *Proc Natl Acad Sci U S A* 2005; **102**(21): 7426-31.

6. Coifman RR, Lafon S. Geometric harmonics: A novel tool for multiscale out-of-sample extension of empirical functions. *Appl Comput Harmon Anal* 2006; **21**(1): 31-52.
7. Giannakis D, Majda AJ. Nonlinear Laplacian spectral analysis for time series with intermittency and low-frequency variability. *Proc Natl Acad Sci U S A* 2012; **109**(7): 2222-7.
8. Coifman R, Lafon S. Diffusion Maps. *Appl Comput Harmon Anal* 2006; **21**: 5-30.
9. Lafon S, Keller Y, Coifman RR. Data fusion and multicue data matching by diffusion maps. *IEEE transactions on pattern analysis and machine intelligence* 2006; **28**(11): 1784-97.
10. Fung R, Hanna AM, Vendrell O, et al. Dynamics from noisy data with extreme timing uncertainty-Supplementary Information. *Nature* 2016; **532**(7600): 471-5.
11. Coifman RR, Shkolnisky Y, Sigworth FJ, Singer A. Graph laplacian tomography from unknown random projections. *IEEE Transactions on Image Processing* 2008; **17**(10): 1891-9.
12. Aubry N, Guyonnet R, Lima R. Spatiotemporal Analysis of Complex Signals - Theory and Applications. *J Stat Phys* 1991; **64**(3-4): 683-739.
13. Villar J, Altman DG, Purwar M, et al. The objectives, design and implementation of the INTERGROWTH-21st Project. *BJOG* 2013; **120 Suppl 2**: 9-26.
14. Villar J, Papageorghiou AT, Pang R, et al. The likeness of fetal growth and newborn size across non-isolated populations in the INTERGROWTH-21st Project: the Fetal Growth Longitudinal Study and Newborn Cross-Sectional Study. *Lancet Diabetes Endocrinol* 2014; **2**(10): 781-92.
15. Villar J, Cheikh Ismail L, Victora CG, et al. International standards for newborn weight, length, and head circumference by gestational age and sex: the Newborn Cross-Sectional Study of the INTERGROWTH-21st Project. *Lancet* 2014; **384**(9946): 857-68.
16. Cheikh Ismail L, Bishop DC, Pang R, et al. Gestational weight gain standards based on women enrolled in the Fetal Growth Longitudinal Study of the INTERGROWTH-21st Project: a prospective longitudinal cohort study. *BMJ* 2016; **352**: i555.
17. Papageorghiou AT, Ohuma EO, Altman DG, et al. International standards for fetal growth based on serial ultrasound measurements: the Fetal Growth Longitudinal Study of the INTERGROWTH-21st Project. *Lancet* 2014; **384**(9946): 869-79.
18. Papageorghiou AT, Kennedy SH, Salomon LJ, et al. International standards for early fetal size and pregnancy dating based on ultrasound measurement of crown-rump length in the first trimester of pregnancy. *Ultrasound Obstet Gynecol* 2014; **44**(6): 641-8.
19. Villar J, Giuliani F, Bhutta ZA, et al. Postnatal growth standards for preterm infants: the Preterm Postnatal Follow-up Study of the INTERGROWTH-21st Project. *Lancet Glob Health* 2015; **3**(11): e681-91.
20. Group WHOMGRS. WHO Motor Development Study: windows of achievement for six gross motor development milestones. *Acta Paediatr Suppl* 2006; **450**: 86-95.

21. Villar J, Cheikh Ismail L, Staines Urias E, et al. The satisfactory growth and development at 2 years of age of the INTERGROWTH-21st Fetal Growth Standards cohort support its appropriateness for constructing international standards. *Am J Obstet Gynecol* 2018; **218**(2S): S841-S54 e2.
22. Villar J, Fernandes M, Purwar M, et al. Neurodevelopmental milestones and associated behaviours are similar among healthy children across diverse geographical locations. *Nat Commun* 2019; **10**(1): 511.
